# PTH-Independent CaSR Inhibition with the Oral Calcilytic Encaleret Improves Calcium Homeostasis in Post-Surgical Hypoparathyroidism: A Phase 2 Open-Label Proof-of-Concept Trial

**DOI:** 10.21203/rs.3.rs-9499053/v1

**Published:** 2026-05-11

**Authors:** Iris Ruth Hartley, Rachel Ilana Gafni, Xiaobai Li, Ashna Grover, Elizabeth A. Ferguson, Casey A. Moore, Karen A. Pozo, Kimberly T. Ampuero, Ananth V. Sridhar, Arun S. Mathew PharmD, Mary Scott Roberts, Scott H. Adler, Edward F. Nemeth, Kelly L. Roszko, Michael T. Collins

**Affiliations:** 1 National Institute of Dental and Craniofacial Research, NIH, Bethesda, MD, USA; 2 Biostatistics and Clinical Epidemiology, Clinical center, NIH, Bethesda, MD, USA; 3 Calcilytix Therapeutics, Inc, San Francisco, CA, USA; 4 MetisMedica, Toronto, ON, Canada.; 4 MT Collins Consulting, Washington, DC, USA

## Abstract

The calcium-sensing receptor (CaSR) maintains calcium homeostasis by regulating parathyroid hormone (PTH) secretion and directly modulating renal calcium reabsorption. In the kidney, PTH and renal CaSR signaling exert opposing effects on tubular calcium reabsorption and urinary calcium excretion, complicating efforts to define their independent contributions to overall calcium regulation. To examine the PTH-independent effects of CaSR inhibition on renal calcium handling, we administered a calcilytic (CaSR antagonist) to individuals with post-surgical hypoparathyroidism (PSH).

In this open-label, phase 2, proof-of-concept study (NCT0573515), ten adults (26-69y) with chronic PSH were orally administered the calcilytic encaleret for up to 5 days. Calcitriol was discontinued one day prior, and calcium and calcitriol were individually titrated based on blood calcium. Mean fractional excretion of calcium (FECa) decreased by 39±29% (p=0.002) by end of treatment.

Albumin-corrected serum calcium increased from 8.5±0.3 mg/dL (mean±SD, normal 8.4-10.2) at time 0 to 9.2±0.6 (p=0.002) at 48 hours while urinary calcium decreased from 405±200 mg/day (m:<250; f:<350) to 180±100 (p=0.0005). Although mean intact PTH increased modestly 30 minutes after the first encaleret dose (7.3±3.1 pg/mL to 12.3±7.9 pg/mL; normal 15-65; p=0.008), levels rapidly returned to baseline without further excursions for the remainder of the study. No serious adverse events (AEs) were reported. Transient mild hypercalcemia and headache in 1 participant were the only treatment-related AEs.

In PSH, encaleret reduced urinary Ca and increased serum Ca, demonstrating the PTH-independent role of CaSR signaling in renal calcium handling and supporting further study of encaleret as an oral treatment for patients with PSH.

## Introduction

Blood calcium concentration is maintained within a narrow physiologic normal range primarily through the coordinated actions of parathyroid hormone (PTH) and the calcium-sensing receptor (CaSR). In response to changes in extracellular calcium concentrations, the CaSR regulates PTH synthesis and secretion in the parathyroid glands and renal calcium reabsorption in the renal tubules.^1^ Decreased CaSR signaling results in increases in circulating PTH which acts to raise blood calcium through multiple mechanisms, including stimulation of bone resorption, increased renal calcium reabsorption, and enhanced intestinal calcium absorption via the increased synthesis of 1,25(OH)2 Vitamin D (1,25D).^2,3^

Within the kidney, both the CaSR and PTH contribute to the regulation of renal calcium reabsorption and urinary calcium excretion. PTH exerts its effects via PTH receptors (PTH1R) located in the thick ascending limb (TAL) and distal tubule, where it increases calcium reabsorption. Although the CaSR is expressed along multiple segments of the nephron, its most clearly defined functional role is in the TAL. In this segment, PTH1R and the CaSR work antagonistically to regulate paracellular calcium reabsorption by modulating claudin protein expression.^3^ The overall effect of PTH1R signaling in the TAL is to promote calcium reabsorption, while CaSR activation promotes calcium excretion. Due to overlapping sites of action and interdependent regulatory pathways, understanding the relative and hierarchical contributions of PTH signaling and direct renal CaSR signaling on overall renal calcium handling has been challenging.

Hypoparathyroidism, a rare disorder of mineral metabolism characterized by hypocalcemia and inadequate PTH levels, provides a unique opportunity to directly investigate human extra-parathyroid CaSR physiology independent of PTH. Post-surgical hypoparathyroidism (PSH), resulting from the inadvertent removal or damage of parathyroid tissue during anterior neck surgery, offers a relatively stable state of absent endogenous PTH secretion.

**Figure 1:**
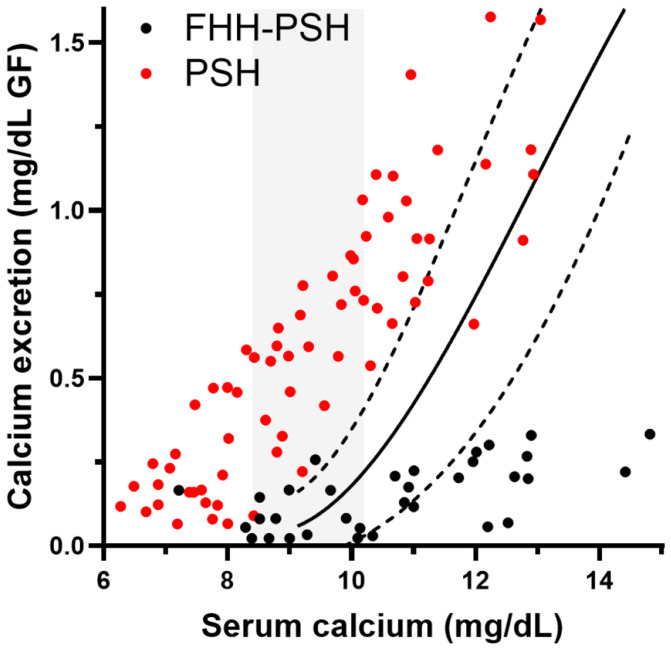
Relationship between serum calcium and urinary calcium excretion The normal calcium excretion curve (mean±2SD, solid and dotted black lines) demonstrates the steep physiologic rise in urinary calcium excretion above a serum calcium threshold of approximately 9 mg/dL. Patients with PSH (red circles) exhibit a leftward shift in this relationship, with the steep rise in urinary calcium occurring at a lower serum calcium threshold (~7 mg/dL). In patients with both FHH and concurrent PSH (black circles), however, the calcium excretion response is markedly attenuated, with minimal increase in urinary calcium across the observed serum calcium range, demonstrating the effect of CaSR inhibition independent of PTH. Gray shaded area denotes the normal serum calcium range. Data adapted from work by Attie et al. and Peacock et al.^5,6^

Under normal physiologic conditions, there is a marked increase in urinary calcium excretion once blood calcium concentrations exceed a defined threshold, quantified by an increase in the fractional excretion of calcium (FECa, Figure 1). In hypoparathyroidism, the loss of PTH action at the renal tubule alters this relationship such that the slope of the calcium excretion curve relating blood and urinary calcium remains largely unchanged, but the curve is shifted to the left. As a result, the steep increase in renal calcium excretion occurs at lower blood calcium levels.^3-6^

The leftward shift in the renal calcium excretion curve has direct implications for the clinical management of hypoparathyroidism. While conventional therapy with oral calcium and active vitamin D analogs can raise blood calcium and alleviate hypocalcemic symptoms, urinary calcium excretion increases disproportionately.^6,7^ Consequently, conventional treatment strategies typically target low to low-normal blood calcium levels, often accepting mild to moderate hypocalcemic symptoms, to minimize the risk of renal complications such as nephrolithiasis, nephrocalcinosis, and chronic kidney disease.^8-10^ The balance between hypocalcemia, hypercalciuria, and symptomatology often presents a significant clinical challenge.

Calcilytics, CaSR antagonists, offer an opportunity to examine how modulation of CaSR signaling influences calcium handling. In individuals with intact parathyroid glands, calcilytics have been shown to increase PTH secretion and raise blood calcium while decreasing urinary calcium excretion.^11-13^ In these populations, the observed increases in renal calcium reabsorption in response to calcilytics reflects a combined effect of increased circulating PTH and direct inhibition of renal CaSRs.

Whether inhibition of renal CaSR signaling alone, independent of PTH, is sufficient to meaningfully alter renal calcium handling in humans has not been directly examined. Rodent studies suggest that reduced CaSR signaling increases renal calcium reabsorption and raises blood calcium independent of PTH.^14-16^ Human data supporting this are limited but informative. In 1983, Attie et al. studied patients with heterozygous loss-of-function variants of the *CASR* (familial hypocalciuric hypercalcemia, FHH1) and concurrent PSH, a clinical situation analogous to patients with PSH treated with a calcilytic. During calcium loading, these individuals demonstrated markedly lower urinary calcium excretion compared to patients with PSH alone (Figure 1). Notably, the normally steep slope of the calcium excretion curve was flattened, even in the presence of overt hypercalcemia and absence of PTH.^5^ Similarly, patients with biallelic loss-of-function variants in *CASR* (neonatal severe hyperparathyroidism, NSHPT) who undergo therapeutic total parathyroidectomy and become hypoparathyroid, are reportedly able to achieve normal blood calcium on conventional therapy with a relatively low risk of hypercalciuria, although published biochemical data in this rare population are limited.^5,17,18^

Based on these preclinical and clinical data, we hypothesized that decreasing CaSR signaling with calcilytics will decrease FECa and urinary calcium excretion and raise blood calcium in the absence of PTH. To test this, we administered encaleret, an oral investigational calcilytic to individuals with PSH and measured the PTH-independent effects of CaSR inhibition on renal calcium handling.

## Results

**Figure 2:**
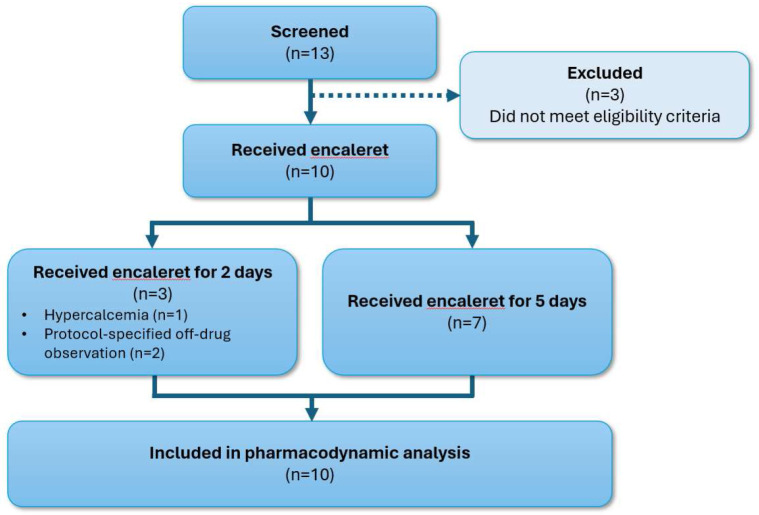
Participant Flow Diagram

Ten participants with PSH, 9 female and 1 male, with a mean age 49 years (range 26-69) completed the study (Figure 2). Three additional female participants were screened but did not meet eligibility criteria due to exclusionary comorbidities. Baseline characteristics and biochemistry of the ten treated participants with PSH are provided in Table 1. Participants had biochemical findings consistent with hypoparathyroidism managed with calcium and calcitriol. Five participants had iPTH less than 10 pg/mL over the course of the study (“aparathyroid cohort”), while the other five experienced at least one measurement of iPTH greater than 10 pg/mL (“residual parathyroid cohort”).

Of the ten participants who completed the study, seven received 5 days (10 doses) of twice daily encaleret (“5-day cohort”). The three remaining participants received 2 days (4 doses) of encaleret (“2-day cohort”). Of these three, one participant discontinued encaleret due to overt hypercalcemia. The two others were selected to discontinue encaleret early to observe the duration of effects following drug discontinuation after they met both the primary and secondary endpoints.

### Encaleret decreased FECa, increased blood calcium, and decreased urine calcium

**Figure 3:**
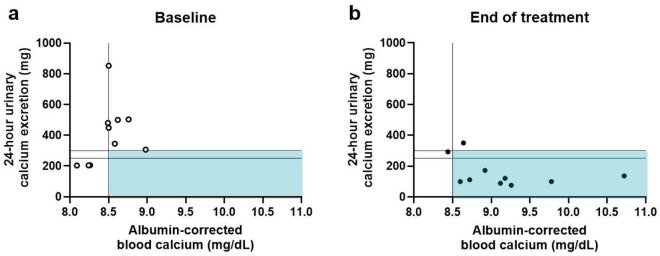
Relationship Between 24-Hour Urinary Calcium and Corrected Serum Calcium at Baseline and End of Treatment. 24-hr UCa excretion versus cCa at baseline (a) and end of treatment (b) show change in this relationship as a result of encaleret. Horizontal lines represent 250 mg/day and 300 mg/day (upper limits of normal [ULN] for women and men, respectively). The vertical line represents the lower limit of normal (LLN) for albumin-corrected serum calcium (8.5 mg/dL). The blue shaded region indicates the target range in which urinary calcium is within the normal range and serum calcium is ≥8.5 mg/dL. At baseline, no patients were within this target region. At the end of treatment timepoint, eight patients had values within the shaded area.

FECa decreased by 39±29% (p=0.002) on encaleret, when comparing values on the final day of treatment to day −1, the prespecified primary endpoint, suggesting that CaSR inhibition significantly altered the relationship between blood and urinary calcium in individuals with PSH. The secondary endpoint, defined as the proportion of participants achieving both normal or elevated fasting albumin-corrected serum calcium (cCa) levels, defined as cCa>8.5 mg/dL, and normal 24-hour urine calcium excretion (24h-Uca, normal range: women <250 mg, men <300 mg) *at any time* during the 5-day admission, was met by 9 participants (90%; 95%CI: 55.5%-99.7%). The remaining participant missed the prespecified endpoint due to a fasting cCa of 8.2 mg/dL on the day his 24h-Uca fell within the normal reference range. Notably, none of the participants met this criterion at baseline on conventional therapy (Figure 3).

**Figure 4:**
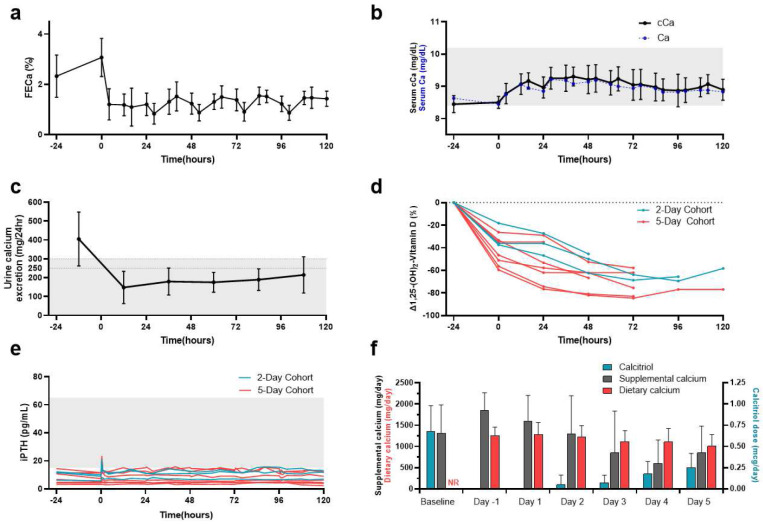
Effects of encaleret on calcium homeostasis. (a) Fractional excretion of calcium (FECa) declined rapidly following the first dose of encaleret (time 0) and remained persistently suppressed. This response resulted in an increase in blood calcium (b) albumin-corrected serum calcium (cCa) in black, total serum calcium in blue) and a marked reduction in urinary calcium excretion (c). (d) Levels of 1,25-dihydroxyvitamin D (1,25D) decreased after discontinuation of calcitriol, with no clear effect attributable to encaleret. 1,25D values are shown only up to the time of calcitriol re-initiation for each individual participant (5-day cohort in red, 2-day cohort in blue). (e) Individual iPTH responses to encaleret revealed a transient peak 30 minutes after the initial dose, followed by levels that remained near baseline thereafter (5-day cohort in red, 2-day cohort in blue). (f) Calcium and calcitriol intake at baseline and during the inpatient study period. Calcitriol was discontinued on day −1 and subsequently supplemental calcium (gray) and calcitriol (blue) were individually titrated. Dietary calcium intake (red) was not quantified at baseline but remained relatively stable on encaleret. Gray shaded regions indicate normal reference ranges. NR = Not Reported. Data is presented as Mean±95% confidence intervals when summarized; Individual data is included in supplemental materials.

Additional analyses were performed to further characterize the impact of encaleret on calcium metabolism. Mean FECa decreased by 63±14% from 3.1±1.1% at 0h to 1.2±0.9% at 4h, the earliest point at which FECa was evaluated (p=<0.0001, Figure 4A). Suppression of FECa persisted at all subsequent on-treatment timepoints. At 48h, the final timepoint at which all participants received encaleret, mean FECa remained low at 1.2±0.6% (p=0.0004). In the 5-day cohort receiving continuous therapy, mean FECa remained suppressed at 1.3±0.4% at the final timepoint (120h, Sup. Fig.1).

The observed reduction in calculated FECa reflects an increase in blood calcium and decrease in urinary calcium. Mean blood cCa increased from 8.5±0.3 mg/dL at 0h to 8.8±0.4 mg/dL at 4h (p=0.009) and 9.2±0.6 mg/dL at 48h (p=0.002, Figure 4B). In the 5-day cohort, cCa at 120h remained elevated above baseline at 9.0±0.5 mg/dL (Sup. Fig.1).

24h-Uca demonstrated a similarly rapid and sustained response to encaleret. Mean 24h-Uca decreased from 405±200 mg at baseline to 148±120 mg (p<0.0001) on day 1 and remained reduced at 180±100 mg on day 2 (p=0.0005, Figure 4C). In the 5-day cohort, mean 24h-Uca remained suppressed throughout treatment, with a day 5 value of 175±105 mg (Sup. Fig. 1).

Eight participants (80%) achieved and maintained 24h-Uca levels within the normal range for the entire treatment period. The remaining two participants, both in the 5-day cohort, achieved 24h-Uca values within the normal range during only a single 24-hour collection period. However, in both individuals, urinary calcium excretion remained substantially reduced from baseline throughout the 5-day treatment period, with reductions in 24h-Uca of 29-56% and 47-66%. Interestingly, these two participants were among the three individuals with the lowest baseline PTH measures (PTH < 5 pg/mL). The third participant in this subgroup, however, maintained consistently low 24h-Uca values (<150 mg/day) across all 5 days of treatment.

After encaleret discontinuation in the 2-day cohort, suppressed FECa gradually increased towards baseline (Sup.Fig.1). Consistent with these changes in FECa, blood cCa decreased and 24h-Uca increased incrementally. Despite the trend towards baseline, mean fasting cCa and 24h-Uca remained within the normal range for 48h after the last dose of encaleret, demonstrating a prolonged pharmacodynamic effect of encaleret after discontinuation.

**Figure 5:**
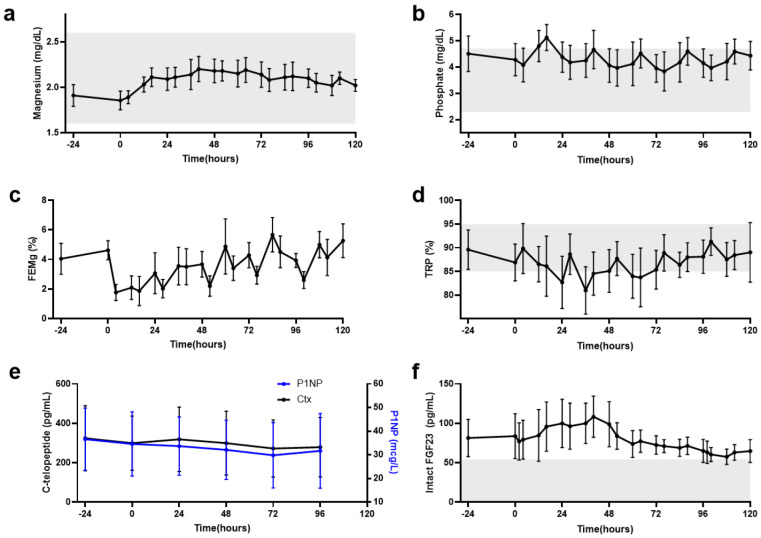
Effects of encaleret on magnesium and phosphate homeostasis and bone turnover (a, c) Serum magnesium concentrations increased while FEMg decreased transiently then returned to baseline values after encaleret treatment. (b) Serum phosphate concentrations showed no significant changes over time. (d, f) Change in TRP and iFGF23 was not significant, however there was a possible trend towards lower TRP and higher iFGF23 during days 2 and 3. (e) Serum bone turnover markers, including procollagen type 1 N-terminal propeptide (P1NP) and C-terminal telopeptide of type I collagen (CTX), showed no clinically meaningful changes from baseline. (f) Data are presented as mean ± 95%CI. Individual data is included in supplemental materials.

One participant experienced an elevation in blood cCa to 10.7 mg/dL requiring early encaleret discontinuation (Sup.Fig. 5). Following discontinuation, blood cCa remained stably elevated, even when supplemental calcium was discontinued and dietary calcium limited. Despite hypercalcemia, concurrent 24h-Uca values were maintained within the normal range for several days. On day 5, more than 60 hours after the final dose of encaleret, blood cCa decreased, accompanied by increases in FECa and 24hr-Uca.

### Calcium and calcitriol intake were lower than baseline on encaleret

Calcium and calcitriol were individually titrated to maintain cCa levels in the normal range (Figure 4F). During the treatment phase of the study, the mean dose of elemental calcium supplementation progressively decreased from 1850±580 mg/day on day −1 to 850±883 mg/day on day 5 (p=0.006). Daily dietary calcium intake, determined by a nutritionist, was 1252±287 mg/day at baseline and did not significantly change while on encaleret. Calcitriol was discontinued on day −1 and reinitiated in response to hypocalcemia or increasing calcium requirements in 7 participants with a mean dose of 0.25±0.24 mcg/day by day 5, less than half the mean pre-admission dose of 0.68±0.43 mcg/day. Three participants were able to maintain cCa within or slightly above the normal range over the final 72 hours of admission in the absence of calcitriol and with minimal calcium supplementation (<500 mg over 72hrs).

### PTH levels were largely unchanged on encaleret

Blood iPTH levels were unchanged during treatment except for a transient spike immediately after the initial dose of encaleret, which did not recur with subsequent doses (Figure 4E). Mean iPTH increased from 7.3±3.0 pg/mL to 12.3±7.9 pg/mL from pre-dose (time 0) to 30 minutes after the first encaleret dose (p=0.008). iPTH levels at peak still remained low or low-normal in all participants with the maximum iPTH measured in any participant of 21 pg/dL. The increase in PTH level was transient, and there were no comparable acute increases after subsequent doses. The iPTH peak was primarily observed in participants in the residual parathyroid cohort. Participants in the aparathyroid cohort experienced minimal (<2.5 pg/mL) or no increase in iPTH at the 30-minute timepoint. In the residual parathyroid cohort, the magnitude of PTH peak strongly correlated with blood cCa at the time of drug administration (r=0.99, p=0.002, Supplemental Fig 3).

Apart from the transient increase observed at 30 minutes, iPTH remained near baseline levels throughout the study. Excluding the 30-minute timepoint, the mean difference between all post-dose iPTH measurements (n=272) and the −24h baseline was 0.006±1.23 pg/ml. Urinary cAMP levels, measured daily while fasting, were also not significantly changed at 48h and 120h compared to baseline, further suggesting that overall encaleret did not change PTH signaling in this population.

All but three participants restarted calcitriol during the study period, limiting our evaluation of endogenous 1,25D production. Excluding measurements obtained after calcitriol reinitiation, 1,25D concentrations trended down and plateaued at a stably low level, consistent with discontinuation of calcitriol prior to admission (Figure 4D).

As the CaSR impacts the reabsorption of other divalent cations at the renal tubules, we also explored the effect of encaleret on blood and urinary magnesium. Serum magnesium had a modest but significant rise during encaleret treatment. Mean serum magnesium increased from 1.9±0.2 mg/dL pre-dose, to 2.2±0.2 at 48 hours (p<0.0001, Figure 5A) and remained mildly increased from baseline at 120 hours at 2.0±0.1 mg/dL (p = 0.004). FEMg decreased from 4.6±0.9% to 1.8±0.8% at 4 hours (p<0.0001) but returned to baseline by 48 hours and 120 hours (Figure 5C).

Parameters of phosphate homeostasis, including blood phosphate, TRP, and intact FGF23 (iFGF23), did not significantly change following encaleret administration although trends suggest a possible transient increase in iFGF23 accompanied by a decrease in TRP without a meaningful change in blood phosphate (Figure 5B, D, F). Serum bone turnover markers, P1NP and CTX, showed no clinically meaningful changes from baseline (Figure 5E). P1NP had a statistically significant decrease at timepoint 48h compared to 0h (p=0.02), but the change in P1NP from 35±19 to 32±18 mcg/L was considered not clinically meaningful.

#### Subgroup analysis:

Subgroup analysis was performed comparing encaleret’s effect on mineral metabolism in the aparathyroid cohort to the residual parathyroid cohort. Changes in FECa, cCa, 24h-Uca, 1,25D, serum magnesium, FEMg, serum phosphate, TRP, FGF23, Ctx, and P1NP at timepoint 48h relative to time 0h showed no significant differences between the cohorts.

### Safety and tolerability

Encaleret was well tolerated with no severe or serious adverse events reported during the trial. 33 mild-moderate AEs (26 mild, 7 moderate) occurred in the 10 participants who received treatment (Supplemental Table 1). 28 AEs were treatment-emergent. Two AEs were considered treatment-related and occurred in one participant during a single event: hypercalcemia and headache. Following these treatment-related adverse events, the protocol was modified to allow for individualized dosing of calcium and calcitriol for safety purposes. All other adverse events were considered unrelated to treatment.

## Discussion

In this phase 2, proof-of-concept study, we investigated the PTH-independent effects of CaSR inhibition in ten individuals with PSH using the calcilytic encaleret. Our findings confirmed our hypothesis that renal CaSR inhibition alone is sufficient to decrease FECa, increase blood calcium levels, and reduce urinary calcium excretion. Urinary Ca effects were rapid and marked with a mean decrease in FECa of 63±14% at 4 hours, and a decrease in mean 24hr-Uca excretion from 405±200 mg/24hr to 148±120 mg on day 1; 24hr-Uca excretion remained suppressed while on treatment. Mean cCa was maintained at a higher level than baseline while on encaleret despite lower calcium and calcitriol intake than prior to treatment. In the early discontinuation group, FECa, 24hr-Uca, and cCa slowly returned to baseline, although 24hr-Uca levels and cCa remained in the normal range at least 2 days after discontinuation, suggesting a prolonged drug effect. Notably, nine of ten participants achieved simultaneously normal or mildly elevated blood calcium and normal 24hr urine calcium excretion. Eight participants maintained a 24hr-Uca level in the normal range for the entire period receiving encaleret; all participants had clear reductions in urinary calcium.

Consistent with preclinical rodent studies and clinical observations in patients with inactivating *CASR* variants,^5,14,15^ our findings provide additional direct clinical evidence that CaSR inhibition has a prominent role in renal calcium handling independent of PTH. Although preliminary, these results further suggest that encaleret may enable patients with PSH to achieve concurrent normalization of blood and urinary calcium, a balance that is difficult to attain with conventional therapy.

We observed a small PTH increase after the first encaleret dose that did not recur with repeated dosing. Although encaleret induced a similar PTH peak 30-minutes post-dose in patients with ADH1, PTH continued to be stimulated with subsequent dosing in that population likely due to intact parathyroid glands.^12^ In the current study, PTH peaks occurred primarily in participants with evidence of residual parathyroid tissue, and were uniformly low in magnitude, with maximum iPTH concentration across all participants and timepoints of only 21 pg/mL. These findings suggest that, in some patients, parathyroid remnants may retain functional calcium-sensing and a limited PTH secretory reserve, although this reserve is rapidly depleted in response to persistently decreased CaSR signaling. Importantly, following the very brief and low magnitude peak after the first dose, PTH levels remained near baseline for the duration of study, enabling us to examine PTH-independent CaSR effects as intended.

Although all participants responded to encaleret, the two with the lowest baseline PTH levels were the only ones whose 24-hr-Uca were above the upper limit of normal while on encaleret. This observation may indicate that a small amount of PTH secretion is necessary to fully optimize renal calcium reabsorption. As the CaSR primarily acts at the TAL of the nephron, inhibition at this site may not fully compensate for the loss of PTH-mediated calcium reabsorption at the distal tubule.^3^ Notably, encaleret still resulted in significant reductions in 24-hr-UCa and FECa in both of these individuals.

Encaleret treatment also resulted in a modest increase in blood magnesium accompanied by a transient reduction in fractional excretion of magnesium, though these effects were less pronounced than observed for calcium. These findings are consistent with the established role of the CaSR in magnesium homeostasis.^4,5^

A subset of participants showed a mild transient increase in iFGF23, with corresponding decrease in TRP after receiving encaleret, but minimal changes in blood phosphate. These changes were variable in timing, duration, and magnitude, and did not meet statistical significance. Our study also showed an apparent lack of effect of encaleret on bone turnover markers, suggesting that calcilytics may not impact bone turnover in the absence of PTH, although definitive conclusions are limited by the short study duration and small sample size.

Although optimization of concomitant therapy was not possible in this short proof-of-concept study, the mean calcium and calcitriol doses during encaleret treatment were lower than at baseline, despite achieving higher mean cCa levels. In fact, three participants treated with encaleret maintained cCa within or slightly above the normal range during the final 72 hours of admission without any calcitriol and with minimal calcium supplementation (<500 mg over 72hrs), demonstrating the marked effect of encaleret. Future studies are needed to determine whether some individuals with PSH can be treated chronically with encaleret monotherapy, or whether all patients would eventually require supplemental calcitriol and/or calcium. Regardless, our findings suggest that supplementation requirements during encaleret therapy are likely to be lower than at baseline.

An additional exploratory goal of this study was to assess whether CaSR inhibition influences 1,25D production. Preclinical data suggested that 1-alpha-hydroxylase activity in vitro and 1,25D production in vivo rapidly increase in the setting of hypocalcemia independently of PTH, presumably via the CaSR.^19,20^ Our data, though limited by the reinitiation of calcitriol in the majority of participants, suggest that this is probably not the case in humans. In the presence of CaSR inhibition, 1,25D levels decreased over the first 48 hours of the study, reflecting calcitriol discontinuation, then plateaued at a low level, and only substantially increased if calcitriol was restarted.

We could not determine whether encaleret enhances the calcemic action of calcitriol, as was suggested by a study in which CaSR disruption in PTH-deficient mice was associated with increased calcemic response to calcitriol.^21^ If pharmacologic CaSR inhibition produces similar effects in humans, patients on encaleret may respond more robustly to calcitriol, requiring lower doses. Further studies are needed to clarify the role, if any, of the CaSR in the regulation of 1,25D synthesis and action.

Encaleret was well tolerated overall, with only two adverse events attributed to the drug, both of which were related to a single episode of hypercalcemia with headache. Hypercalcemia was prevented in all subsequent participants following protocol revision that permitted individualized calcium dosing.

The main limitations of our study were small study size and short duration of exposure. The substantial magnitude and consistency of the response in blood and urinary calcium to encaleret provided sufficient statistical power for primary outcomes, but the small sample size limited meaningful assessment of some potential effects of CaSR inhibition. The brief intervention period also did not allow for optimization of supplemental calcitriol and/or calcium therapy or evaluation of long-term efficacy and safety.

Another limitation is the potential for fluctuating calcitriol administration to have influenced our results by impacting intestinal calcium absorption and blood calcium levels. However, decreasing 1,25D levels following calcitriol discontinuation would be expected to work in the opposite direction of the observed changes. It would have decreased intestinal calcium absorption, resulting in higher calcium supplement requirements and lower blood calcium during the observation period. Thus, our conclusions regarding encaleret’s effect on calcium homeostasis remain valid. Future studies administering encaleret with stable calcitriol dosing could better isolate the more subtle effects of CaSR modulation on mineral regulation.

Additional trials across diverse etiologies of hypoparathyroidism are needed to validate and expand these findings and establish long-term safety and efficacy of encaleret in hypoparathyroidism.

Future studies may also test an intriguing possibility: whether chronic calcilytic therapy could promote residual parathyroid tissue proliferation and restore parathyroid function. Notably, calcimimetics, CaSR-activating drugs, reduce parathyroid mass in patients with secondary hyperparathyroidism, suggesting that calcilytics, with the opposite physiologic effect, might stimulate parathyroid growth.^22,23^ Preclinical data regarding calcilytic’s impact on parathyroid tissue is limited and mixed. Lim et al. observed possible improvement in functional recovery of autotransplanted parathyroid tissue in calcilytic-treated hypoparathyroid rats.^15^ However, prolonged treatment with the calcilytic NPS2143 for 8 weeks did not increase parathyroid cell proliferation in osteopenic rats.^24^ Moreover, many patients with PSH are chronically maintained at low blood calcium levels on conventional therapy, which would be expected to result in persistently reduced CaSR signaling; yet late restoration of parathyroid function is rarely observed. Nevertheless, whether the high drug exposure tolerated by patients with PSH could stimulate parathyroid proliferation during prolonged therapy or promote recovery shortly after parathyroid injury remains an important question warranting further investigation. Other potential applications of calcilytics, such as treatment of idiopathic hypercalciuria, could be explored in the future if a dose can be identified that reduces urinary calcium without inducing hypercalcemia.

In conclusion, this study demonstrates that CaSR inhibition in humans increased renal calcium reabsorption independent of the action of PTH. Further clinical investigation is warranted to determine whether CaSR inhibition can be used for the treatment of hypoparathyroidism.

## Materials and methods:

This study was an investigator-sponsored, open-label, proof-of-concept phase 2 trial (NCT05735015). It was approved by the Institutional Review Board of the National Institute of Dental and Craniofacial Research (NIDCR). Written informed consent was obtained from all participants. It was conducted in accordance with the principles established by the Declaration of Helsinki.

### Participants:

Adults with a clinical diagnosis of PSH were eligible for participation in this study. Patients were excluded from the treatment phase of the study if they had *severe* symptoms despite normal or low-normal blood calcium levels, hypocalcemic seizures within the previous 3 months, treatment with any PTH analogs within the previous 3 months, 25OH-vitamin D <25 ng/mL or >60 ng/mL, or any risk factors for osteosarcoma. Additional exclusion criteria included comorbid conditions such as active cardiac disease, anemia, and insufficient hepatic or renal function (eGFR<50 mL/min/1.73m2). Participants with clinically significant active abnormalities in thyroid function tests were excluded, however, participants with stable thyroid replacement therapy and on TSH-suppression for thyroid cancer were eligible. Full inclusion and exclusion criteria are included in the supplemental materials.

### Trial Design

After a screening evaluation, eligible participants were admitted to the NIH Clinical Center for a week-long admission. After baseline assessments, encaleret 162mg was administered orally every 12 hours to participants for up to 5 days. This dose was one of the highest doses evaluated in a prior study of encaleret in ADH1^12^. A high dose was selected to minimize the risk of a type II error due to insufficient drug exposure. Encaleret was supplied by Calcilytix Therapeutics, Inc. Since this was a proof-of-concept study, after meeting the primary and secondary endpoints, encaleret was stopped in select participants to observe the off-drug recovery effects on mineral metabolism.

### Concomitant calcium and activated vitamin D

Participants were asked to consume approximately 1000 mg of calcium daily, although their dietary intake was not strictly restricted. With the assistance of a nutritionist, participants maintained a dietary journal and actual calcium intake was quantified daily.

Calcitriol was stopped one day prior to initiation of encaleret to observe the impact of CaSR inhibition on 1,25D production. The protocol was initially written to provide a robust standard calcium load, however, after the first participant became hypercalcemic, the protocol was modified to allow individual titration of calcium and calcitriol to maintain normal blood calcium at the discretion of the investigator. On day 3 or after, if calcitriol had not yet been restarted, participants were given calcitriol if corrected calcium remained <8.5 mg/dL, to be assured all subjects had an adequate filtered load of calcium to detect a change in FECa. Magnesium supplementation was stopped at least 2 days prior to initiation of encaleret, and thiazide diuretics were discontinued greater than 5 drug half-lives prior to admission.

After the treatment phase, participants’ calcium and calcitriol were restarted at doses individualized to the participant. Follow up safety biochemical assessments occurred 3 to 7 days after the last dose of encaleret, and participants were remotely monitored for adverse events as outpatients for 30 additional days.

### Biochemical and imaging assessments:

Serial blood samplings via intravenous catheter and urine sampling, including 24-hour urine collections, were performed throughout the admission. Participants fasted from midnight to 8 AM until they received the morning encaleret dose and had pre-dose blood and urine sampling.

Routine blood and urine chemistries were performed at the NIH Clinical Center Department of Laboratory Medicine. Additional biochemical assessments included iPTH (electrochemiluminescence immunoassay on Roche Cobas e601 analyzer; NIH Clinical Center, Bethesda, MD, USA), 1,25D (chemiluminescent immunoassay on DiaSorin Liaison XL; NIH Clinical Center), intact FGF23 (enzyme-linked immunosorbent assay, Immutopics International, San Clemente, CA, USA), serum collagen C-telopeptide (CTx; electrochemiluminescence immunoassay Elecsys Beta-CrossLaps Roche Diagnostics; Mayo Medical Laboratories, Rochester, MN, USA), and serum procollagen Type 1 N-propeptide (P1NP, competitive radioimmunoassay UniQ P1NP RIA Orion Diagnostica; Mayo Medical Laboratories).

FECa was determined from blood samples and paired interval urine collections using the calculation: FECa = (urine calcium*serum creatinine) / (urine creatinine*serum calcium)*100%. TRP was calculated using the following calculation: TRP = (1- (urine phosphate*serum creatinine)/(urine creatinine*serum phosphate))*100%. Renal ultrasound was performed at baseline to screen for nephrocalcinosis and nephrolithiasis.

### Study objectives and assessments:

The pre-specified primary efficacy endpoint was the percent change in fractional excretion of calcium from conventional therapy baseline (Day −1) to the final day of treatment (Day 6 or the last available on-treatment fasting assessment). The secondary endpoint was proportion of participants who achieved cCa > 8.5 mg/dL with concomitant normal 24-hour urine calcium excretion (<250 mg/24h for women, <300 mg/24h for men) during the 5-day admission.

For other mineral metabolism measures, analyses were performed using the 4h, 24h, 48h and 120h timepoints, each compared with baseline at 0h. Bone turnover markers were last measured at 96h; therefore, this timepoint was used in place of 120h for those measures. Due to the observed peak in iPTH, iPTH was also analyzed at the timepoint 30 minutes after daily morning encaleret doses.

### Statistical analysis

All statistical analyses and graphical outputs were generated using GraphPad Prism version 10 (GraphPad Software, Inc., La Jolla, CA, USA) and confirmed with SAS version 9.4 (SAS Institute Inc. Cary, NC, USA). Laboratory values below the lower limit of detection were imputed as one-half of the lower limit. 24h urine assessments with excretion values unable to be calculated due to concentrations lower than the lower limit of normal, were imputed as 0 mg. Data are presented as mean±standard deviation (SD) at each timepoint.

Data normality was assessed using the Shapiro–Wilk test. For normally distributed data, one-sample or two-sample paired t-tests were used to compare timepoints. For non-normally distributed data, the Wilcoxon signed-rank test was used.

To analyze the secondary endpoint, the proportion of participants achieving the specified criteria, the percentage along with its 95% confidence interval were calculated using the exact binomial distribution.

Subgroup analyses were conducted based on residual parathyroid function. Participants were classified as “aparathyroid” if all iPTH values were <10 pg/mL, and “residual parathyroid” if at least one iPTH value exceeded 10 pg/mL during the study. For between-group comparisons, data normality was assessed using the Shapiro-Wilk test. When normally distributed, unpaired t-tests were used, with equal variances confirmed by an F test.

Since this is a small, early-phase trial with a small number of subjects, no multiplicity adjustment was applied to the p-values. A two-sided p-value less than 0.05 was considered statistically significant.

Figure 1 was adapted from original data reported by Attie et al. and Peacock et al.^5,6^ Data was extracted from published figures using WebPlotDigitizer.^25^ For normal individuals, mean and ±2SD of calcium excretion were calculated across serum calcium increments of 0.25 mg/dL between 9 and 10 mg/dL, 0.5 mg/dL between 10 and 11 mg/dL, and 1 mg/dL between 11 and 15 mg/dL. Third order polynomial curves were fitted to the mean and ±2SD values with high goodness of fit (R^2^ >0.99). For individuals with PSH, data combined from Attie et al. and Peacock et al. were included; PSH-FHH data were from Attie et al.

## Supplementary Material

Supplementary information accompanies the manuscript on the Bone Research website http://www.nature.com/boneres

This is a list of supplementary files associated with this preprint. Click to download.

- PSHsupplement.pdf
- NIHManuscriptCoverSheet.pdf

## Figures and Tables

**Table 1.** Baseline Characteristics

Demographics and Clinical History	Value (n=10) n(%); mean±SD (range)
Age (years)	49±13 (26-69)
Female	9 (90%)
Time since surgery (years)	6±4 (1-12)
Indication for surgery	PTC - 6 (60%) MNG - 3 (30%) MNG/microPTC - 1(10%)
Renal ultrasound findings	Nephrocalcinosis - 2 (20%) Nephrolithiasis - 1 (10%) Both - 1 (10%)
Baseline Regimen	
Elemental Calcium, mg/day	1314±928 (85-3600)
Calcitriol, mcg/day	0.68±0.43 (0.25-1.5)
Biochemistry (Blood)	
Corrected-Calcium, mg/dL	8.4±0.4 (7.9-9.3)
Magnesium, mg/dL	1.9±0.2 (1.7-2.2)
Phosphate, mg/dL	4.5±0.9 (3.2-5.9)
eGFR, mL/min/1.73 sq.m	80±16 (59-113)
iPTH, pg/dL	8.6±4.0 (3.2-14.5)
25(OH) Vitamin D, ng/mL	45±5 (38-52)
1,25(OH)_2_ Vitamin D, pg/mL	31±12 (11-47)
TSH, mcIU/mL	1.8±0.5 (0.2-2.1)
Biochemistry (Urine)	
Calcium Excretion, mg/24hr	405±200 (204-853)
FECa, %	2.3±1.2 (0.8-4.5)
TRP, %	90±6 (79-96)
Cyclic AMP Excretion, nmol/dL	1.8±0.5 (1.1-2.5)

Abbreviations: Papillary Thyroid Cancer (PTC), Multinodular Goiter (MNG), Thyroid Stimulating Hormone (TSH), Tubular Reabsorption of Phosphate (TRP).

Normal ranges: Albumin-corrected calcium: 8.4-10.2 mg/dL, Magnesium: 1.6-2.6 mg/dL, phosphate: 2.5-4.5 mg/dL, iPTH: 15-65 pg/mL, 25(OH) Vitamin D: 33-100 ng/mL, 1,25(OH)_2_ Vitamin D: 19-79 pg/mL. TSH: 0.35-4.94 mcIU/mL, urinary calcium excretion: <250 mg/24hr (female), <350 mg/24hr (male), TRP: 85-95%, cyclic AMP excretion 1.3-3.7 nmol/dL. Normal range for FECa is not defined, but values in euparathyroid, eucalcemic patients are typically <2%.

## Data Availability

De-identified data underlying the results reported in this manuscript will be made available by the corresponding author upon reasonable request.
